# CGP57380 enhances efficacy of RAD001 in non-small cell lung cancer through abrogating mTOR inhibition-induced phosphorylation of eIF4E and activating mitochondrial apoptotic pathway

**DOI:** 10.18632/oncotarget.8497

**Published:** 2016-03-30

**Authors:** Qiuyuan Wen, Weiyuan Wang, Jiadi Luo, Shuzhou Chu, Lingjiao Chen, Lina Xu, Hongjing Zang, Mohannad Ma Alnemah, Jian Ma, Songqing Fan

**Affiliations:** ^1^ Department of Pathology, the Second Xiangya Hospital, Central South University, Changsha, Hunan, 410011, China; ^2^ Cancer Research Institute of Central South University, Changsha, Hunan, 410078, China

**Keywords:** non-small cell lung cancer (NSCLC), mTOR, eIF4E, Mnk1, apoptosis

## Abstract

The mammalian target of rapamycin (mTOR) is a potentially important therapeutic target in a broad range of cancer types. mTOR inhibitors such as rapamycin and its analogs (rapalogs) have been proven effective as anticancer agents in non-small cell lung cancer (NSCLC), whereas they strongly enhance phosphorylation of eukaryotic translation initiation factor 4E (eIF4E) and activation of Akt, which cause resistance to mTOR-targeted therapy after an initial response. Rapamycin induces eIF4E phosphorylation by activating MAPK-interacting kinases (Mnks), and therefore targeting Mnk/eIF4E pathway represents a potential therapeutic strategy for the treatment of NSCLC. Here, our results showed that over-expression of p-Mnk1 and p-eIF4E was significantly associated with poor overall survival of NSCLC patients and high expression of p-Mnk1 might act as an independent prognostic biomarker for these patients. Meanwhile, inhibiting Mnk1 expression by Mnk inhibitor (CGP57380) could abrogate rapalogs (RAD001)-induced eIF4E phosphorylation and Akt activation. Furthermore, combination of CGP57380 and RAD001 could induce NSCLC cells apoptosis via activating intrinsic mitochondrial pathway, and exert synergistic antitumor efficacy both *in vitro* and *in vivo*. In conclusion, combination of targeting both mTOR and Mnk/eIF4E signaling pathways to enhance effectiveness of mTOR-targeted cancer therapy might be significant innovation for the personalized treatment of NSCLC.

## INTRODUCTION

Nowadays, with the recognition of EGFR and KRAS gene mutations, MET amplification and EML4-ALK rearrangements, new therapies directed against defined molecular targets profoundly impact on the non-small cell lung cancer (NSCLC) [1]. The PI3K/AKT/mTOR signaling is an important intracellular signaling pathway involved in the regulation of multiple cellular functions including cell proliferation, survival, differentiation, adhesion, motility and invasion [2, 3]. Recent evidences suggest that PIK3/AKT/mTOR signaling is frequently activated in NSCLC and plays important role in the oncogenesis. Inhibition of these pathways contributes to enhance the therapeutic efficacy of EGFR-targeted drugs in the treatment of NSCLC [4, 5].

Mammalian target of rapamycin (mTOR), a kinase in the PI3K/AKT/mTOR signaling pathway, integrates growth factor stimulation with energy and nutrient signaling to control cell growth and proliferation, which plays a key role in cell growth, protein translation, autophagy, angiogenesis and metabolism [6]. Inhibitors of mTOR such as rapamycin and its analogs can inhibit the function of mTOR, leading to inactivation of ribosomal S6K1 and inhibition of cap-dependent translation initiation through the 4E-BP1/eIF4E pathway. Unfortunately, several studies showed that rapamycin actually increased phosphorylation of eukaryotic translation initiation factor 4E (eIF4E) and activation of Akt, which is closely associated with development of cell resistance to mTOR inhibitors and will weaken the anticancer efficacy of the mTOR inhibitors [7–9].

eIF4E has a key regulatory role in initiating mRNA translation [10], which is known to be up-regulated in a variety of human cancers and is linked to poor prognosis [11]. eIF4E induces cell proliferation, evasion of apoptosis, angiogenesis and metastasis through enhancing translation of a subset of mRNAs involved in oncogenesis such as cyclinD1, c-Myc, Mcl-1, Bcl-2, VEGF, MMP9 and so on [12, 13]. During translation initiation, eIF4E activity can also be modified by MAPK-interacting kinases Mnk1 and Mnk2, which are activated by Erk and p38 and phosphorylated eIF4E on S209 via banding and in concert with the assembly of the eIF4F complex [14–16]. Phosphorylation of eIF4E is elevated in a wide variety of human cancers and is a prognostic marker in lung cancer [17, 18]. Since mTOR inhibitor induced phosphorylation of eIF4E in a Mnk-dependent manner, combination of mTOR inhibitor and Mnk inhibitor is a promising strategy to improve the efficacy of mTOR inhibitor in human cancers [8, 19]. Indeed, combined pharmacologic inhibition of mTOR and Mnk kinases has shown highly effective on suppressing tumor growth in prostate cancer cells, glioma and lymphoma cells [20–23].

In the present study, our findings showed that overexpression of p-Mnk1 and p-eIF4E was significantly associated with poor overall survival of NSCLC patients and high expression of p-Mnk1 could serve as a poor prognostic biomarker for these patients, so targeting Mnk/eIF4E pathway for blocking Mnk function and eIF4E phosphorylation might be a potential therapeutic strategy for treatment of NSCLC patients, particularly for these patients with overexpression of p-eIF4E and p-Mnk1. Furthermore, we found that Mnk inhibitor CGP57380 could abrogate RAD001-activated eIF4E phosphorylation, and the combination of Mnk inhibitor and mTOR inhibitor could augment the antitumor efficacy through inhibiting proliferation and inducing apoptosis in NSCLC cells. Therefore, combination of targeting both mTOR signaling and Mnk/eIF4E pathway to enhance mTOR-targeted cancer therapy might be an innovation therapeutic strategy for NSCLC patients.

## RESULTS

### P-Mnk1 and p-eIF4E expression increases and correlates with poor prognosis in NSCLC

**Figure 1:**
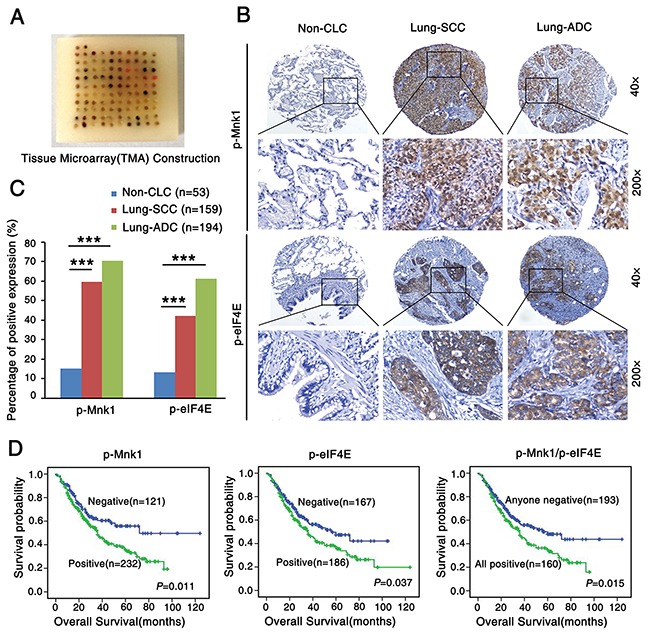
P-Mnk1 and p-eIF4E expression increases and correlates with poor prognosis in NSCLC **A.** Tissue microarray (TMA) construction for 53 cases of non-cancerous lung tissues (Non-CLT) and 353 cases of non-small cell lung cancer (NSCLC) including 159 cases of lung squamous cell carcinoma (SCC) and 194 cases of lung adenocarcinoma (ADC). **B.** Representative immumohistochemical staining of p-Mnk1 and p-eIF4E in Non-CLT, lung SCC and ADC tissue using special antibodies. p-Mnk1 was predominantly localized in the nucleus and p-eIF4E was predominantly expressed in the cytoplasm (magnification 200× and 40×). **C.** Expression of p-Mnk1 and p-eIF4E in lung SCC and ADC compared to Non-CLT. Results showed that there were significant differences between the groups which were statistically evaluated by chi-square test (***P < 0.001). **D.** Kaplan-Meier analysis was used to plot the overall survival curves of 353 cases of NSCLC patients with different expression of p-Mnk1, p-eIF4E and combined expression of these two proteins, which statistical significance was assessed by log-rank test. NSCLC patients with positive expression of p-Mnk1, p-eIF4E and common positive expression of these two proteins showed worse overall survival rates compared to patients with negative p-Mnk1, p-eIF4E and negative either of these two proteins (*P* = 0.011, *P* = 0.037, *P* = 0.015, two sided, respectively).

To investigate the correlation between expression of p-Mnk1 and p-eIF4E and clinicopathological features of NSCLC, expression of p-Mnk1 and p-eIF4E was detected by immunohistochemistry (IHC) in the NSCLC tissue microarray (TMA) (Figure 1A) which contained 353 cases of NSCLC and 53 cases of non-cancerous lung tissues (Non-CLT). Positive expression and distribution of p-Mnk1 and p-eIF4E were identified in the nucleus and cytoplasm of lung squamous cell carcinoma (SCC) and lung adenocarcinoma (ADC) tissues, respectively. And no staining was found in the Non-CLT (Figure 1B). Positive percentage of both p-Mnk1 and p-eIF4E expression was significantly higher in lung SCC and ADC tissues compared to the Non-CLT (*P* < 0.001) (Figure 1C). The correlation between expression of p-Mnk1 and p-eIF4E and clinicopathological features of NSCLC was shown in Supplementary Table S2. There was significantly positive correlation between over-expression of p-Mnk1 and the histological types of NSCLC. Importantly, lung ADC had significantly higher expression of p-Mnk1 than that of lung SCC (*P* = 0.032). The similar situation was also observed in the expression of p-eIF4E in these cases (*P* < 0.001). In addition, NSCLC patients with positive expression of p-Mnk1 (*P* = 0.001), p-eIF4E (*P* = 0.003) as well as common positive of these two proteins (*P* < 0.001) had more short overall survival times than those with negative expression of these proteins mentioned above. The further analysis of the pair-wise association showed that expression of p-Mnk1 was significantly positive associated with that of p-eIF4E in the NSCLC(r = 0.451, *P* < 0.001, spearman rank correlation test) (Supplementary Table S3).

Furthermore, the results from Kaplan-Meier survival curve analysis with log-rank significance test showed that the overall survival rate for NSCLC patients with negative expression of p-Mnk1 was significantly higher than those with positive p-Mnk1 expression (*P* = 0.011), as well as the overall survival rate for NSCLC patients with negative expression of p-eIF4E was better than these with positive p-eIF4E expression (*P* = 0.037) (Figure 1D). In addition, NSCLC patients with common positive expression of p-Mnk1 and p-eIF4E had a lower survival rate than patients with any negative staining of two proteins above (*P* = 0.015) (Figure 1D). Moreover, multivariate Cox's proportional hazard regression analysis indicated that the positive expression of p-Mnk1 could act as an independent poor prognostic biomarker for NSCLC patients (*P* = 0.035), regardless of lymph node metastasis (LNM) status, clinical stages and pathological grades (*P* = 0.04, *P* < 0.001, *P* = 0.01, respectively) (Table 1). The multivariate model, however, did not confirm the prognostic significance of patients' age, gender, histological type, treatment strategy and the expression of p-eIF4E in NSCLC (*P* > 0.05, respectively).

**Table 1:** Summary of multivariate analysis of Cox proportional hazard regression for overall survival in 353 cases of NSCLC patients

Parameter	B	S.E.	Wald	Sig.	Exp(B)	95.0%CI for Exp(B)	
						Lower	Upper
**Age**	0.113	0.176	0.410	0.522	1.120	0.792	1.582
**Gender**	−0.233	0.201	1.349	0.245	0.792	0.534	1.174
**Histological types**	0.201	0.170	1.408	0.235	1.223	0.877	1.706
**LNM status**	0.402	0.196	4.207	0.040*	1.495	1.018	2.194
**Treatment strategy**	0.026	0.190	0.019	0.891	1.026	0.708	1.489
**Clinical stages**	0.691	0.197	12.331	0.000*	1.996	1.357	2.936
**Pathological grades**	0.435	0.168	6.696	0.010*	1.545	1.111	2.148
**p-Mnk1**	0.443	0.211	4.432	0.035*	1.558	1.031	2.353
**p-eIF4E**	0.027	0.185	0.153	0.696	1.075	0.748	1.546

Abbreviations: LNM, lymph node metastasis; CI, confidence interval.

Note: multivariate analysis of Cox regression, *P < 0.05.

### Combination of targeting both mTOR signaling and Mnk/eIF4E pathway inhibits the proliferation of NSCLC cells

**Figure 2:**
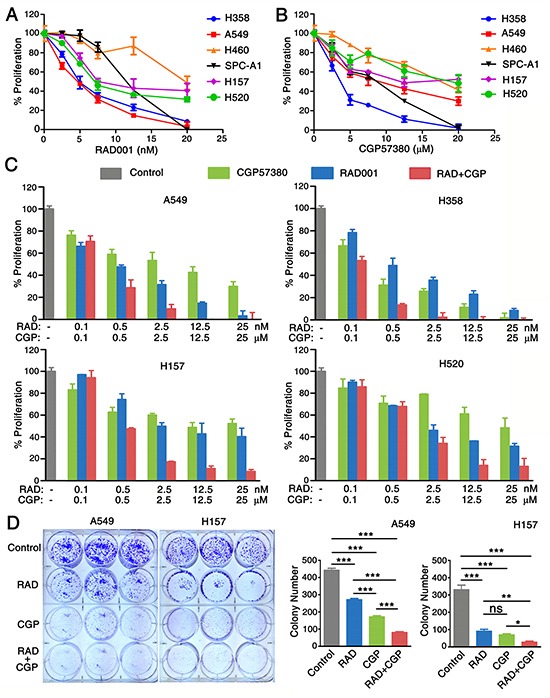
Combination of targeting both mTOR signaling and Mnk/eIF4E pathway inhibits the proliferation of NSCLC cells **A.** A panel of human NSCLC cell lines as indicated were seeded in 96-well plates and then treated with different concentrations of RAD001 (RAD) ranging from 0.1 to 20nM. **B.** CGP57380 (CGP) ranging from 0.1 to 20μM as indicated on the second day. After 3 days, cell numbers were estimated using SRB assay. Points, means of triplicate determinations; mean ± SD. **C.** A SRB-based proliferation assay of the indicated cell lines after 3 days of treatment with CGP57380 (CGP) alone or treatment with RAD001 (RAD). Results were obtained in triplicate and are shown as % proliferation compared with control cells. Columns, means of three replicate determinations; each bar represents, mean ± SD. **D.** A549 and H157 cells were seeded in 6-well plates at a density of 1000 cells per well. On the second day, the cells were treated with 0.5nM RAD001 (RAD) alone, 6.5μM CGP57380 (CGP) alone, and their combination. The same treatment was repeated every 3 days. After 10 days, the plates were stained for the formation of cell colonies with crystal violet. The colonies in each well were counted. The bars are means of three replicate determinations plus standard deviations. NS, non-significant, * *P* < 0.05, ** *P* < 0.01, ***P < 0.001.

RAD001 (everolimus), a derivative of rapamycin, is an orally bioavailable mTOR inhibitor tested in clinical trials. CGP57380 is a novel low-molecular-weight kinase inhibitor of Mnk [24]. In this study, we conducted a 3-day cell survival assay to identify the effects of RAD001 and CGP57380 on inhibiting the proliferation of human lung cancer cells *in vitro*. All NSCLC cell lines except human large-cell lung cancer cell line NCI-H460 (H460) involving in our study were sensitive to RAD001 (Figure 2A) and CGP57380 (Figure 2B). In order to investigate whether combined targeting mTOR and Mnk/eIF4E pathway would result in augmenting growth-inhibitory effects on lung cancer cells, four human NSCLC cell lines were treated by RAD001 alone or in combination with CGP57380. As presented in Figure 2C and 2D, in a 3-day SRB assay and a 10-day colony formation assay, the combination of RAD001 and CGP57380 was more efficacy than that of either single-agent on inhibiting proliferation and colony formation of human NSCLC cell lines.

### Concomitant treatment with mTOR inhibitor RAD001 and Mnk1 inhibitor CGP57380 inhibits growth of lung cancer tumor *in vivo*

**Figure 3:**
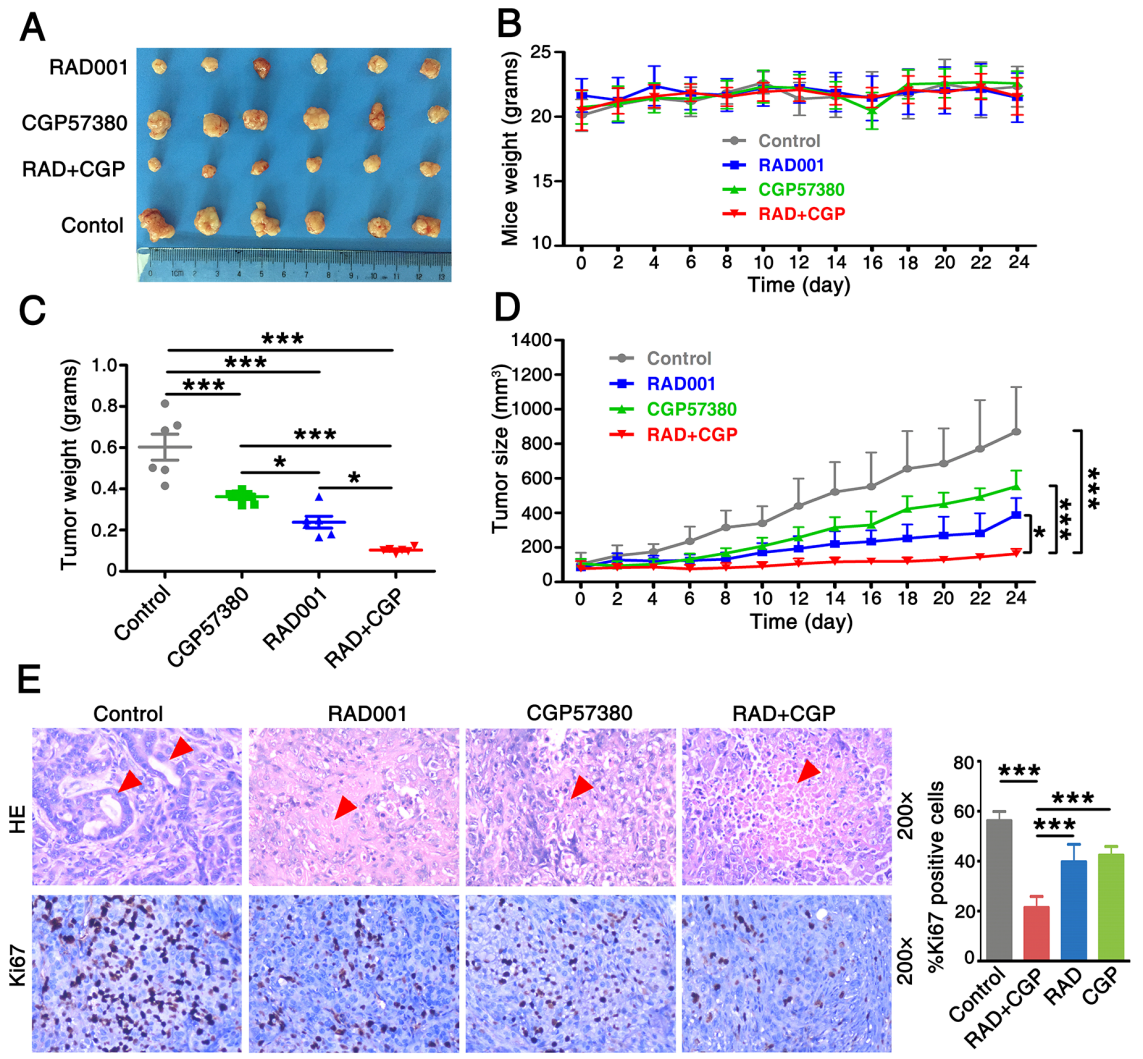
Concomitant treatment with CGP57380 and RAD001 inhibits growth of lung cancer tumor *in vivo* **A.** Xenografts nude mice were treated with vehicle control, RAD001 alone, CGP57380 alone and RAD001 plus CGP57380 on the day after grouping. After 24 days, the mice were sacrificed and the tumors were removed. **B.** The weight of 4 groups nude mice were measured every two days. **C.** The 4 groups' tumor weight was evaluated at indicated time. * *P* < 0.05, *** *P* < 0.001. **D.** Growth curves for an NSCLC xenografts nude mouse model. Tumor volume (mm^3^) was measured using a caliper at the indicated time points. Data are shown as mean ± SD. *P < 0.05, ***P < 0.001. **E.** H&E staining (top) and IHC for Ki-67 (bottom) in control and treated tumors. The percentage of Ki-67 positive cells was determined by counting stained cells in the treated tumors. Each bar represents mean ± SD. ***P < 0.001.

To examine the inhibition effect of combination of CGP57380 and RAD001 on the development and growth of lung cancer *in vivo*, A549 cells were employed in xenograft experiments in BALB/c nude mice. We found that the concomitant treatment with CGP57380 and RAD001 had a markedly greater effect than that of either agent alone on the inhibitor of tumor growth (Figure 3A). Results also showed that concomitant treatment with CGP57380 and RAD001 induced a marked reduction in the volume and weight of lung cancer A549 cell xenografts *in vivo* (Figure 3A, 3C). No obvious toxicity was observed in any groups during the treatments with oral administration of RAD001 and intraperitoneal administration of CGP57380. During the observation period there was no significant difference in the body weight of nude mice among the four groups (Figure 3B).

The relative tumor growth curves for control-, CGP57380-, and/or RAD001-treated animals are shown in Figure 3D. Control tumors showed the exponential growth, the treatment with CGP57380 alone significantly reduced tumor growth (*P* = 0.001), as did RAD001 alone (*P* < 0.001). Combination treatment with CGP57380 and RAD001 led to the most pronounced effect of growth-inhibitory on the A549 cell xenografts (*P* < 0.001). H&E staining showed the formation of gland cavities in tumors and the emergence of massive necrosis in the combination of CGP57380 and RAD001 treatment group. Furthermore, Ki-67 labelling index measured by IHC demonstrated the significantly lowest number of Ki-67 positive cells in this group mentioned above. These results suggested that combination of targeting both mTOR signaling and Mnk/eIF4E pathways exerted an obvious growth inhibition effect on human NSCLC cells (Figure 3E).

### Mnk1 inhibitor CGP57380 abrogates the eIF4E phosphorylation induced by mTOR inhibitor RAD001 both *in vitro* and *in vivo*

**Figure 4:**
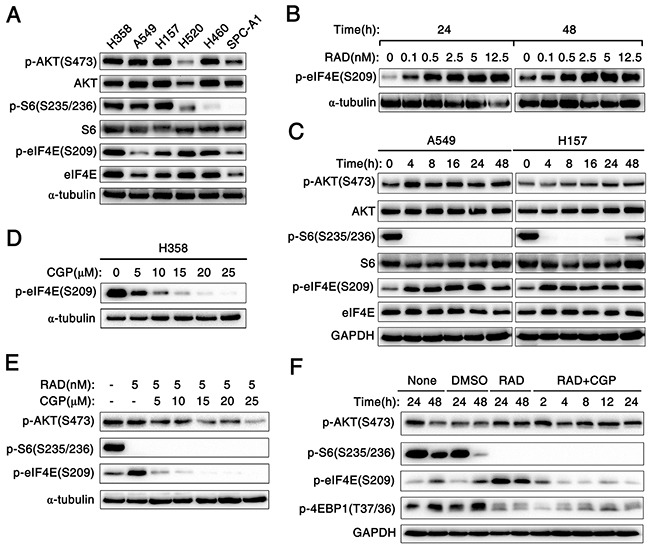
Mnk1 inhibitor CGP57380 abrogates the eIF4E phosphorylation induced by mTOR inhibitor RAD001 *in vitro* **A.** Western blotting analysis for the expression of several proteins including the phosphorylated and total levels of AKT, S6 and eIF4E in six kinds of human NSCLC cell lines. α-tubulin was used as a loading control. **B.** A549 cells were treated with the indicated concentrations of RAD001 (RAD) for 24 h and 48 h. **C.** A549 and H157 cells were treated with 5nM RAD001 (RAD) for the indicated times. **D.** H358 cells were treated with the indicated concentrations of CGP57380 (CGP) for 24 h. **E.** A549 cells were treated with combination of 5nM RAD001 (RAD) and the indicated concentrations of CGP57380 (CGP) for 24 h. **F.** A549 cells were treated with 5nM RAD001 (RAD) for 24 h and then co-treated with DMSO, or 10μM CGP57380 (CGP) for the indicated times. After these treatments, the cells were harvested for preparation of whole-cell protein lysates and subsequent Western blotting analysis to detect the indicated proteins. α-tubulin or GAPDH was used as a loading control.

In this study, human lung cancer cell lines were used to investigate the effect of RAD001 on phosphorylation of eIF4E, the expression of key proteins in the AKT/mTOR signaling pathway such as AKT, p-AKT (S473), S6, p-S6 (S235/236), eIF4E and p-eIF4E (S209) were detected in six human NSCLC cell lines by Western blot. A relatively low expression level of p-eIF4E (S209) was observed in A549, H157 and SPC-A1 cell lines compared to other lung cancer cell lines (Figure 4A). RAD001 significantly induced expression of p-eIF4E in A549 cells even at a concentration of 0.1nM when A549 cells were treated with various concentration of RAD001 for 24h and 48h (Figure 4B). In addition, RAD001 at concentration of 5 nM strikingly increased expression level of p-eIF4E protein in A549 cells, which could rapidly occur after 4 h of treatment with RAD001. Similar trend about p-AKT (S473) was also observed in A549 cells (Figure 4C).

**Figure 5:**
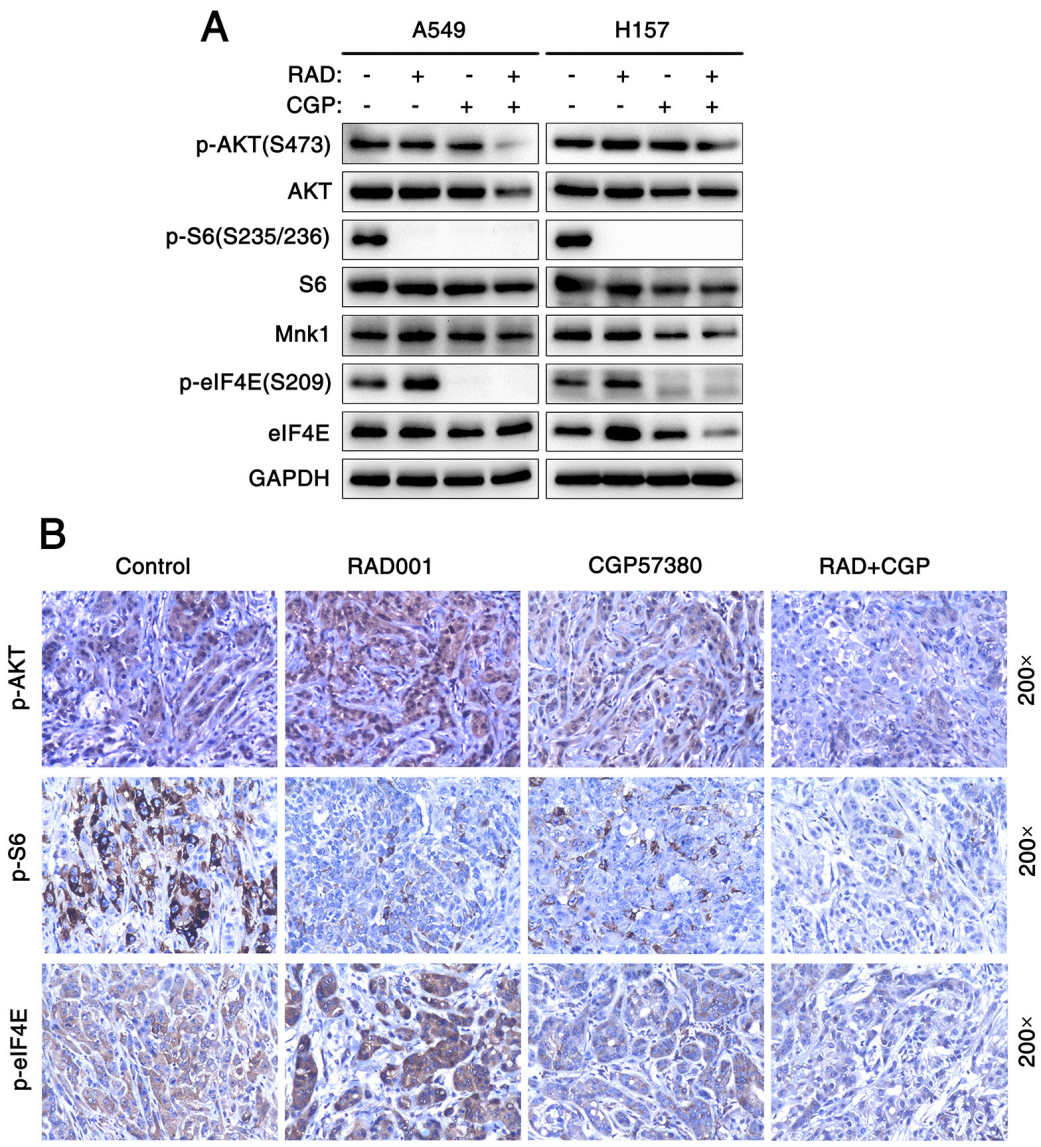
Concomitant treatment with mTOR inhibitor and Mnk inhibitor attenuate eIF4E and AKT phosphorylation both *in vitro* and *in vivo* **A.** Cell lysates were collected from A549 and H157 cells which were treated with 25 nM RAD001 (RAD) alone, 25μM CGP57380 (CGP) alone and both combination for 24 h and subjected to Western blotting for the indicated proteins. GAPDH served as a loading control. **B.** IHC for p-AKT, p-S6 and p-eIF4E in tumor tissues from nude mouse xenograft models of human lung cancer cells.

Furthermore, we examined the effect of CGP57380 on the abrogation of eIF4E phosphorylation. Interestedly, decreased expression of p-eIF4E protein was observed from 5μM concentration to higher concentrations of CGP57380 in H358 cells (Figure 4D). To investigate whether CGP57380 indeed blocked RAD001-induced eIF4E phosphorylation, p-eIF4E was detected in A549 cells treated with RAD001 alone or combination of RAD001 and CGP57380. As shown in Figure 4E and 4F, CGP57380 could inhibit RAD001-induced eIF4E phosphorylation, particularly at an early time of treatment (e.g., 2 h), whereas inhibition of S6 phosphorylation was not affected by RAD001. Thus, these data clearly showed that CGP57380 abrogated RAD001-induced eIF4E phosphorylation, but it did not block RAD001 inhibited the phosphorylation of S6. Besides, we noted that co-treatment of the lung cancer cell lines with RAD001 and CGP57380 resulted in the obviously decrease expression of p-AKT protein at 24 h, at which RAD001-induced eIF4E phosphorylation was substantially inhibited by CGP57380 (Figure 4E and 5A). Moreover, similar trend of expression levels of p-AKT (S473), p-S6 and p-eIF4E proteins in A549 xenografts tissues described above were also detected by IHC (Figure 5B).

### Combination of targeting both mTOR signaling and Mnk/eIF4E pathway induces cell apoptosis both *in vitro* and *in vivo*

**Figure 6:**
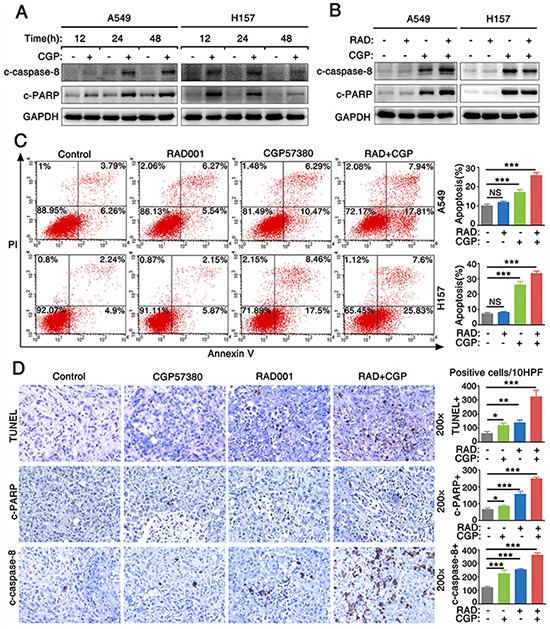
Combination of targeting both mTOR signaling and Mnk/eIF4E pathway induces cell apoptosis both *in vitro* and *in vivo* **A.** Cell lysates were collected from A549 and H157 cells which were treated with 25μM CGP57380 (CGP) for different times as indicated. Then Western blotting was used to detect the indicated proteins and GAPDH was used as a loading control. **B.** A549 and H157 cells were treated with 25nM RAD001 (RAD) alone, 25μM CGP57380 (CGP) alone and their combination for 24 h. Western blotting was used to detect the indicated proteins and GAPDH was used as a loading control. **C.** Apoptotic cells were analyzed by flow cytometry using Annexin V/PI staining. Columns, means of three replicate determinations; each bar represents mean ± SD. NS, non-significant, *** *P* < 0.001. **D.** TUNEL assay and IHC for c-PARP and c-caspase-8 in tumor tissues from A549 xenografts. Positive cells were counted in 10 high-power fields (x400) under a light microscopy, * *P* < 0.05, ** *P* < 0.01 ***P < 0.001. Each bar represents mean ± SD (n = 6 mice/group).

To evaluate whether CGP57380 enhances antitumor efficacy of RAD001 on lung cancer cells through inducing cell apoptosis, we firstly detected the expression level of cleaved-caspase-8 (c-caspase-8) and PARP (c-PARP) in the lung cancer cells treated with CGP57380 and RAD001. The expressions of c-caspase-8 and c-PARP were significantly increased in A549 and H157 cell lines treated with CGP57380 at a concentration of 25 μM even after 24 h or 48 h. Results indicated that CGP573980 could significantly induce NSCLC cells apoptosis (Figure 6A). Furthermore, we investigated whether CGP57380 combined with RAD001 had synergistic apoptosis-inducing effects on NSCLC cells. Results showed that CGP57380 alone or its combination with RAD001 exhibited a much greater potency in inducing apoptosis (Figure 6C) via increasing c-caspase-8 and c-PARP expression (Figure 6B). In addition, apoptosis was measured by in situ end labeling (TUNEL) staining and expressions of c-PARP and c-caspase-8 were detected by IHC staining, results showed A549 xenografts treated with combination of CGP57380 and RAD001 had significantly higher percentages of positive apoptotic cells (Figure 6D). The results suggested that the combination of CGP57380 and RAD001 could exert synergistic apoptosis-inducing effect on NSCLC cells.

### Combination of targeting both mTOR signaling and Mnk/eIF4E pathway induces apoptosis through an intrinsic mitochondrial pathway

**Figure 7:**
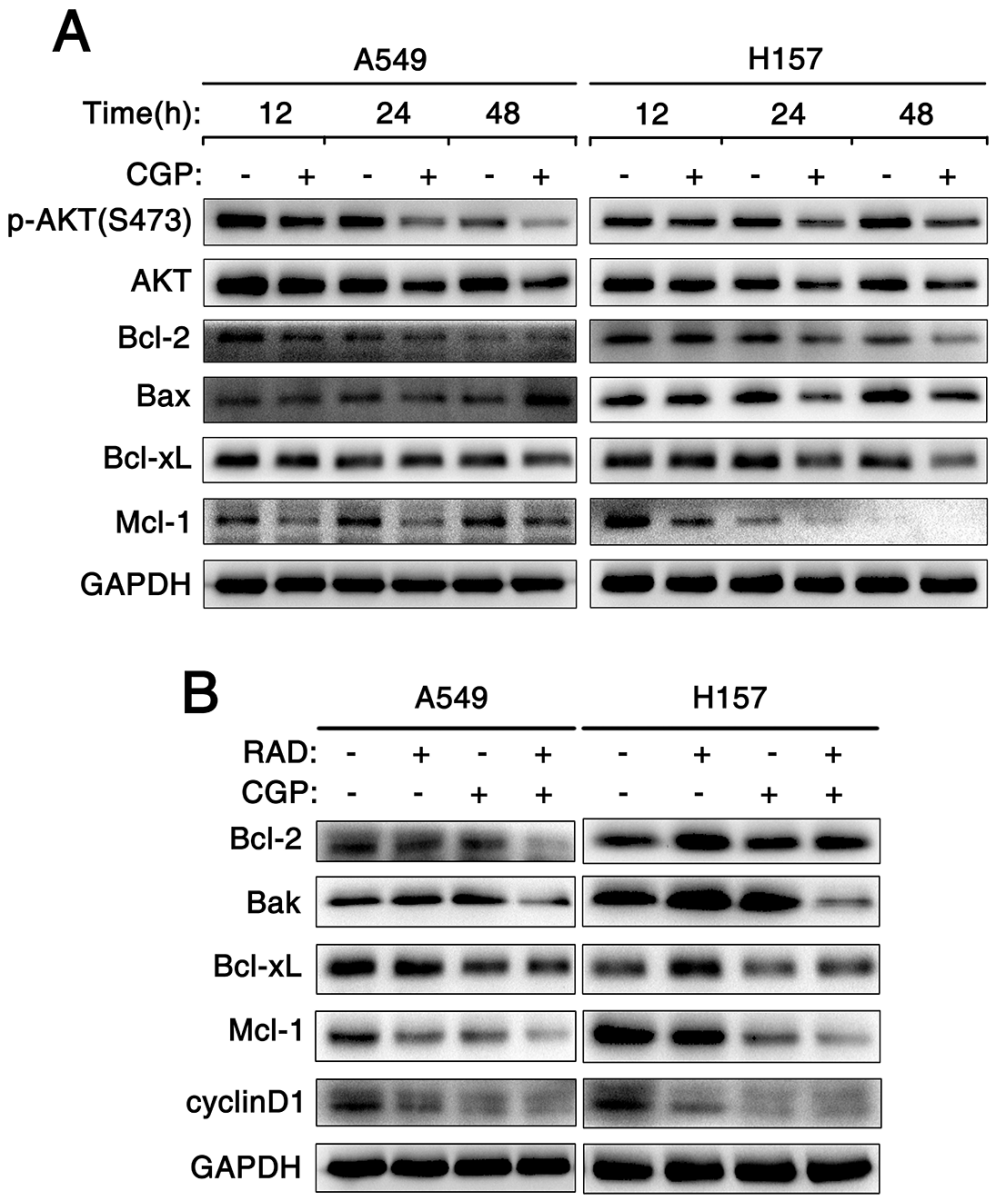
Combination of targeting both mTOR signaling and Mnk/eIF4E pathway induces apoptosis through an intrinsic mitochondrial pathway **A.** A549 and H157 cells were treated with 25μM CGP57380 (CGP) for different times as indicated. Then Western blotting was used to detect the indicated proteins and GAPDH was used as a loading control. **B.** A549 and H157 cells were treated with 25nM RAD001 (RAD) alone, 25μM CGP57380 (CGP) alone and their combination for 24 h. Then cells were harvested for Western blotting analysis to detect the indicated proteins. GAPDH was used as a loading control.

To understand the mechanism of apoptosis induced by CGP57380 alone and that of exerting synergistic apoptosis-inducing effects on NSCLC cells by combination of CGP57380 and RAD001, the key proteins known to be involved in regulation of the extrinsic or intrinsic apoptotic pathway (i.e., death receptors, Bcl-2 family members) were detected by Western blot. Firstly, A549 and H157 cell lines were treated with CGP57380 at different times. Then, western blotting was used to detect Bcl-2, Bcl-xL, Mcl-1 and Bax (Figure 7A). Results indicated that the levels of Bcl-2, Bcl-xL and particularly Mcl-1were obviously reduced in A549 and H157 cell lines after treating by CGP57380 as early as 12 h, these effects were sustained up to 48h. Furthermore, the maintained reduction of protein levels of Bcl-2, Bcl-xL and Mcl-1 was noticed in A549 and H157 cell lines after prolonging treatment with CGP57380. As far as Bax was concerned, it noticeably increased after treating 48 h by CGP57380 in A549, but significantly reduced as early as 12 h in H157 cell (Figure 7A). Therefore, we are especially interested in identifying the major Bcl-2 family members in the following experiments.

NSCLC cell lines were treated with RAD001, CGP57380, and combination of RAD001 and CGP57380 for 24 h and western blot was hired to detect proteins mentioned above. Rather than increasing, the expression levels of DR4, DR5 and Bax proteins decreased in A549 and H157 cell lines (Supplementary Figure S1A and S1B). Even expression of DR4 protein was not been detected in H520 cells (Supplementary Figure S2B). The results suggested that these proteins were not involved in mediating cell apoptosis induced by CGP57380 or in combination with RAD001. Most interestingly, we found that the concomitant treatment of CGP57380 and RAD001 could evidently decrease the expression levels of Bcl-2, Bcl-xL proteins and especially Mcl-1 protein than treatment with either agent alone in A549 and H157 cell lines (Figure 7B). These results further supported the notion that down-regulation of Bcl-2 and Bcl-xL proteins and especially Mcl-1 protein was a critical mechanism by which CGP57380 enhances RAD001-induced apoptosis. In addition, result showed that the expression level of cyclinD1 protein in A549 and H157 treated by the combination of CGP57380 and RAD001 was effectively reduced (Figure 7B). These results suggested that the combination of RAD001 and CGP57380 could exert synergistic antitumor efficacy through inhibiting cell proliferation, and inducing apoptosis via activation of intrinsic mitochondrial pathway in NSCLC cells.

## DISCUSSION

eIF4E is a well-established proto-oncogene, whose expression or activation is associated with transformation and tumorigenesis. Increasing evidences implicate the Mnks and/or Mnk-dependent phosphorylation of eIF4E play important roles in cell transformation, tumorigenesis or tumor progression [25, 26]. The high expression of p-eIF4E has been verified in a variety of human cancers and predicts poor prognosis. Our results showed that expression of p-eIF4E was up-regulated and associated with poor prognosis in NSCLC, which was in a good agreement with previous literatures [18]. Up to now, the associations between the expression of p-Mnk1 and the clinicopathological features and prognostic implications of NSCLC have not been reported. In the present study, over-expression of p-Mnk1 was significantly associated with poor overall survival of NSCLC patients and high expression of p-Mnk1 might act as an independent poor prognostic factor for these patients, which was similar to the findings in our previous study on nasopharyngeal carcinoma (NPC) [27]. So, Mnk/eIF4E pathway may represent an attractive anticancer drug target for the treatment of NSCLC patients. Mnk has emerged as an exciting and promising strategy for cancer therapy, for example, degradation of Mnk1 blocks eIF4E phosphorylation and subsequently inhibits cell growth, colonization, invasion, migration and induces apoptosis in triple negative and HER2-overexpressing breast cancer cell lines [28]. Inhibition of Mnk/eIF4E pathway in blast crisis chronic myeloid leukemia could effectively prevent self-renewal of leukemia stem cells [29].

Semi-synthetic rapamycin analogues with improved pharmacokinetic properties, collectively known as rapalogy, including Temsirolimus (Toricel), Ridaforolimus (MK-8669) and Everolimus (RAD001) [2]. Temsirolimus and ridaforolimus have been shown to exert a strong antitumor activity in NSCLC subcutaneous tumor-bearing mice, and to prolong survival in pleural disseminated tumor-bearing mice, after using a series of intermittent dosing schedules [30, 31]. RAD001 is already use as a post-transplant immunosuppressive agent and has been shown to inhibit the proliferation of tumor cell growth in preclinical studies, and phase I/II clinical trials in lung cancer are under way. In particular, at a daily dose of 10 mg, RAD001 was reported to be active and safe in pretreated advanced NSCLC patients [32]. However, therapy with mTOR inhibitors alone can induce activation of p-eIF4E resulting in enhancing mTOR-targeted therapy resistance. Inducible phosphorylation of eIF4E at Ser-209 mediated by Mnk1 and Mnk1 activation is necessary for phosphorylation of eIF4E. So, combination of mTOR inhibitor with Mnk inhibitor may be an effective therapeutic tactics to enhance the anticancer efficacy and overcome resistance to mTOR inhibitors in NSCLC. In the present study, our data demonstrated that RAD001 combined with CGP57380 exhibited enhanced inhibitory effects on proliferation and colony formation of human NSCLC cells. Also, enhanced induction of apoptosis was found in NSCLC cells treated with combination of CGP57380 and RAD001 *in vitro*. Importantly, the combination of RAD001 and CGP57380 worked better than that of treatments with each single agent in inhibiting the growth of human lung cancer xenografts in nude mice, indicating an enhanced anticancer activity *in vivo* too. Moreover, reduced Ki-67 labelling index in xenografts tissues and expression of cyclinD1 protein was also found in following experiment. These data indicated that combination of CGP57380 to block Mnk/eIF4E pathway might inhibit cell proliferation and induce apoptosis to enhance RAD001′s antitumor efficacy in NSCLC. Our findings pave the way for clinical testing of new rational therapeutic strategies to prevent or overcome resistance to mTOR-targeted cancer therapy in NSCLC.

It is critical to understand the molecular mechanisms of exhibiting enhanced inhibitory effects on NSCLC cells by combination of mTOR and Mnk inhibitors, and which will help us to develop new therapy strategies to overcome resistance in mTOR-targeted cancer therapy. Our data provided convincing evidence that RAD001 could increase eIF4E phosphorylation in several kinds of NSCLC cell lines. Besides inducing eIF4E phosphorylation, activation of Akt was also enhanced in NSCLC cells after treating by RAD001. These results consisted with previous reports that phosphorylation of Akt could be induced by mTOR inhibitor [7, 8]. eIF4E, a translational regulator, acts downstream of Akt and mTOR and recapitulates Akt's action in tumorigenesis and drug resistance *in vivo* [33]. Dual targeting of both Akt and mTOR, or directly inhibiting eIF4E activity has been proposed as treatments for cancer [8, 34, 35]. CGP57380 could substantially reduced eIF4G in the eIF4F complex and drastically inhibited eIF4E phosphorylation [24, 36]. Our results showed that CGP57380 could abrogate RAD001-induced Akt phosphorylation besides attenuate RAD001-induced eIF4E phosphorylation. Thus, these results suggested that CGP57380 could augment the antitumor efficacy by abrogating RAD001-induced eIF4E phosphorylation and inhibiting Akt activation in NSCLC.

The previous investigations indicate that Mnk inhibitor can induce apoptosis in lymphoma and lung cancer, but the specific mechanism of apoptosis has not been elucidated [22, 37, 38]. In the present study, we further investigate the detailed molecular mechanisms by which CGP57380 induces apoptosis to exert augmented antitumor effect on NSCLC cells. It is well known that cells can die of apoptosis primarily through the extrinsic death receptor-induced pathway and/or the intrinsic mitochondria-mediated pathway [39, 40]. DR4 and DR5 are key components in the TRAIL-mediated extrinsic apoptotic pathway [41]. The Bcl-2 protein family which includes Bcl-2, Bcl-xL, Mcl-1, Bax, Bak and so on is key component in intrinsic mitochondria-mediated pathway [42]. Our present results indicated that CGP57380 decreased the expression levels of the anti-apoptotic Bcl-2 family members such as Mcl-1, Bcl-2 and Bcl-xL proteins, but no significant increasing in the expression levels of DR4 and DR5 was observed in all tested cell lines. Meanwhile, significantly lower expression levels of Mcl-1, Bcl-2 and Bcl-xL proteins were observed in the NSCLC cell lines treated with combination of RAD001 and CGP57380. Furthermore, both flow cytometry (FCM) analysis and TUNEL staining demonstrated that combination of RAD001 and CGP57380 could induce NSCLC cell apoptosis. These data suggested that down-regulation of anti-apoptotic Bcl-2 family members (e.g., Mcl-1, Bcl-2 and Bcl-xL) might be a critical event that mediates CGP57380-induced apoptosis in NSCLC cells. Therefore, combination of targeting both mTOR signaling and Mnk/eIF4E pathway may elicit NSCLC cells apoptosis through an intrinsic mitochondria-mediated pathway. Nonetheless, other than the results mentioned above, we found that except cyclinD1, there was no significant difference in the expression levels of p-Akt, Bcl-2, Mcl-1 and Bcl-xL in H520 cell lines treated with RAD001 and CGP57380 alone or the combination of RAD001 and CGP57380. Results suggested that combination of RAD001 and CGP57380 might induce apoptosis via other mechanism in lung SCC. This is an interesting point and its underlying mechanisms are worth further investigation in our future studies.

In summary, there was significantly positive correlation between expression of p-Mnk1 and p-eIF4E proteins in NSCLC tissues. High expression of p-Mnk1 could act as an independent poor prognostic biomarker for NSCLC patients. Furthermore, combination of RAD001 and CGP57380 could exert synergistic inhibitory effect on cell proliferation, colonization as well as growth of lung cancer xenografts by inducing apoptosis via activation of intrinsic mitochondrial pathway. In conclusion, CGP57380 enhances RAD001′s antitumor efficacy in NSCLC involving abrogation of mTOR inhibition-induced eIF4E phosphorylation and activation of intrinsic mitochondrial apoptotic pathway. Combination of targeting both mTOR signaling and Mnk/eIF4E pathway might be significant innovation for personalized therapy in NSCLC patients.

## MATERIALS AND METHODS

### Clinical data and tissue microarrays (TMA) construction

NSCLC patients were submitted to surgical treatment at the Department of Thoracic Surgery at the Second Xiangya Hospital of Central South University (Changsha, China) from 2003 to 2013. All patients had undergone a complete staging examination before definitive treatment. These patients had been effectively treated by surgical resection of both the primary tumor (at least lobectomy) and systematic mediastinal lymph node dissection. All samples were obtained with informed consent and all protocols were approved by the Second Xiangya Hospital of Central South University Ethics Review Board (Scientific and Research Ethics Committee, no. s02/2000). Written informed consent was obtained from all patients, also the written informed consent was obtained from the next of kin, caretakers, or guardians on the behalf of the minors/children participants involved in our study. These patients had a confirmed histological diagnosis of NSCLC according to WHO histological classification of the lung cancer. The staging classification of the current analysis was carried out based on the criteria of the 7th edition of the AJCC/UICC TNM staging system of lung cancer (2009). No patients had been previously treated with chemotherapy and radiotherapy at the time of original operation. Complete clinical record and follow-up data were available for all patients.

Patient characteristics were detailed in Supplementary Table S1. In this study, we used the TMA technology designed and constructed high-throughput NSCLC TMAs according to rules previously described [43, 44].

### Immunohistochemistry and score

Immunohistochemistry (IHC) analysis for p-Mnk1 and p-eIF4E in NSCLC TMA sections and their staining were scored as previously described [43]. The evaluation was based on the staining intensity and extent of staining. Staining intensity for p-Mnk1 and p-eIF4E was scored as 0 (negative), 1 (weak), 2 (moderate), and 3 (strong). Staining extent was scored as 0 (0%), 1 (1-25%), 2(26-50%), 3 (51-75%), and 4 (76-100%), depending on the percentage of positive-stained cells. The sum of the staining intensity and the staining extent scores ranged from 0 to 7, and an optimal cut-off level was identified as follows: a staining index score of 0 was used to define tumors with negative expression and 1-7 indicated positive expression of these two proteins. Agreement between the two evaluators was 95%, and all scoring discrepancies were resolved through discussion between the two evaluators.

### Cell lines and reagents

The human NSCLC lines A549, H358, H157, H460, H520 and SPC-A1 were obtained from the Cell Bank of the Chinese Academy of Sciences (Shanghai, China) where they were characterized by mycoplasma detection, DNA-Fingerprinting, isozyme detection and cell vitality detection. These cell lines were immediately cultured and frozen down such that all cell lines could be restarted every 3-4 months from a frozen vial of the same batch of cells. All cells were cultured in RPMI-1640 (Gibco, USA) medium supplemented with 10% fetal calf serum (Gibco, USA) at 37°C in a humidified 5% CO_2_ incubator. The inhibitors and antibodies were purchased from commercial sources: mTOR inhibitor Everolimus (RAD001) (Selleckchem, Houston, TX, USA), and Mnk1 inhibitor CGP57380 (Sigma-Aldrich, USA). Anti-Akt polyclonal antibody, anti-eIF4E polyclonal antibody, anti-S6 mAb (clone 54D2), anti-p-S6(S235/236) mAb, anti-p-4EBP1(T37/36) mAb (clone 236B4), anti-cyclinD1mAb (clone 92G2), anti-cleaved caspase-8 mAb (clone 18C8), anti-cleaved-PARP polyclonal antibody, anti-Mcl-1 polyclonal antibody, anti-p-Mnk1 (Thr197/202) polyclonal, anti-Bcl-xL mAb (clone 54H6), anti-Bak mAb (clone D4E4) (Cell signaling, MA, USA), Anti-p-Akt (S473) mAb (clone 2109Y), anti-p-eIF4E (S209) mAb, anti-Mnk1 mAb, anti-Bax mAb (Abcam, cambridge, UK), Anti-DR5 polyclonal antibody (ProSci, Inc, Poway, CA, USA); Anti-DR4 mAb (Imgenex, San Diego, CA, USA); anti-Bcl-2 mAb (clone 8C8) (UcaIIM Biotechnology Co, Wuxi, China), Anti-GAPDH mAb, anti-α-tubulin polyclonal antibody (Proteintech Group, CHI, USA).

### Growth inhibition assay

Cells were seeded at densities ranging from 3×10^3^-5×10^3^ cells/well in 96-well cell culture plates and treated with the agents indicated the next day. The viable cell number was determined using the sulforhodamine B (SRB) assay, as previous described [45].

### Colony formation assay

Cells (A549 and H157; single-cell suspension) were cultured in 6-well plate at a density of 1000 cells per well. After 24 h, A549 and H157 cells were treated with RAD001 (0.5 nM), CGP57380 (6.5μM), RAD001+CGP57380, and DMSO as vehicle control, and incubated for 10 days to allow colony formation. Cell culture medium was changed with the corresponding concentration of the agents every 3 day. Colonies containing more than 50 cells were counted and evaluated.

### Detection of cell apoptosis by flow cytometry

Detection of apoptotic cells was done with the FITC Annexin V/ propidium iodide (PI) Apoptosis Detection Kit (BD, Pharmingen, TM). Briefly, 10^6^ A549 and H157 cells were exposed for 24 h to RAD001 (25nM), CGP57380 (25μM), or combination. Washed with phosphate-buffer saline (PBS), and then incubated in the dark at room temperature with Annexin V/FITC and PI for 15 min. The apoptotic cells were enumerated using the flow cytometer (FACS Calibur, BD Biosciences). Annexin V-FITC-positive and PI-negative cells were considered as early apoptotic, whereas positivity for both Annexin V-FITC and PI was associated with late apoptosis or necrosis.

### Western blotting

Whole-cell protein lysates were prepared and analyzed by Western blotting analysis as described previously [45].

### Lung cancer xenografts and treatments

A549 cells (1×10^7^) in 200μl serum-free medium were injected subcutaneously in the right flank of the four-to five-week old female BALB/c nude mice (Slac Laboratory Animal, Shanghai, China). All experimental procedures were approved by the Institutional Animal Care and Use Committee of Central South University and performed in accordance with the Declaration of Helsinki. When tumors reached certain size ranges (50-100mm^3^), the mice were randomized into four groups (n = 6/group) according to tumor volumes for the following treatment: vehicle control, formulated RAD001 (3mg/kg/day, oral gavage), CGP57380 (25mg/kg, three times a week, i.p injection), and the combination of RAD001 and CGP57380. Tumor volumes were measured using caliper measurements once every two days and calculated with the formula V = π (length×width^2^)/6. After a 24-day treatment, the mice were anesthetized and killed by cervical dislocation. The tumors were then removed, weighted, and fixed with formalin. After the tumors were embedded in paraffin, 4μm sections were cut and processed for H&E staining, IHC and TUNEL assay.

### TUNEL assay

TUNEL assays were carried out on the sectioned tissue samples using the In situ cell Death Detection Kit, PoD (Roche, Mannheim, Germany) as the manufacturer's instructions. In brief, tumor tissue sections were deparaffinized and rehydrated and then pretreated with proteinase K (20mg/ml; Roche, Mannheim, Germany) for 20 min at 37°C. The samples were then placed in 3% H_2_O_2_ for 30 min at room temperature. The tumor sections were subsequently incubated with TUNEL reaction mixture (add 50μl Enzyme solution (vial 1) to the remaining 450μl Label solution in vial 2 to obtain 500μl TUNEL reaction mixture) at 37°C for 60 min. The slides were rinsed with PBS and then the tumor sections were incubated with converter-POD for 30 min at room temperature. Color reaction was developed by using 3, 3′-diaminobenzidine tetrachloride (DAB) chromogen solution. All slides were counterstained with hematoxylin for 10 min. The slides were examined and positive cells were counted in 10 random high-power fields (magnification ×400) under a light microscope.

### Statistical analysis

The chi-square test and the Spearman's rank correlation coefficient were used to evaluate the relationship between the p-Mnk1 and p-eIF4E expression in tissue sections harvested from NSCLC patients. Kaplan-Meier analysis was performed for overall survival curves and statistical significance was assessed using the log-rank test. Cox proportional hazard regression model was used to estimate the independent prognostic factor p-Mnk1 and p-eIF4E. Other statistical significant was determined by One-way ANOVA test and the Dunnett-T3-test especially for tumor volume. Two-sided statistical analysis was used and the data were considered to be statistically significant when *P* < 0.05.

## SUPPLEMENTARY FIGURES AND TABLES


