# Supplementary material for: CGP57380 enhances efficacy of RAD001 in non-small cell lung cancer through abrogating mTOR inhibition-induced phosphorylation of eIF4E and activating mitochondrial apoptotic pathway
Source: Oncotarget. 2016 Mar 30;7(19):27787–801. doi: 10.18632/oncotarget.8497 (PMC5053688; doi:10.18632/oncotarget.8497)
Supplement: Supplementary file 1 [file oncotarget-07-27787-s001.pdf]

SUPPLEMENTARY FIGURES AND TABLES

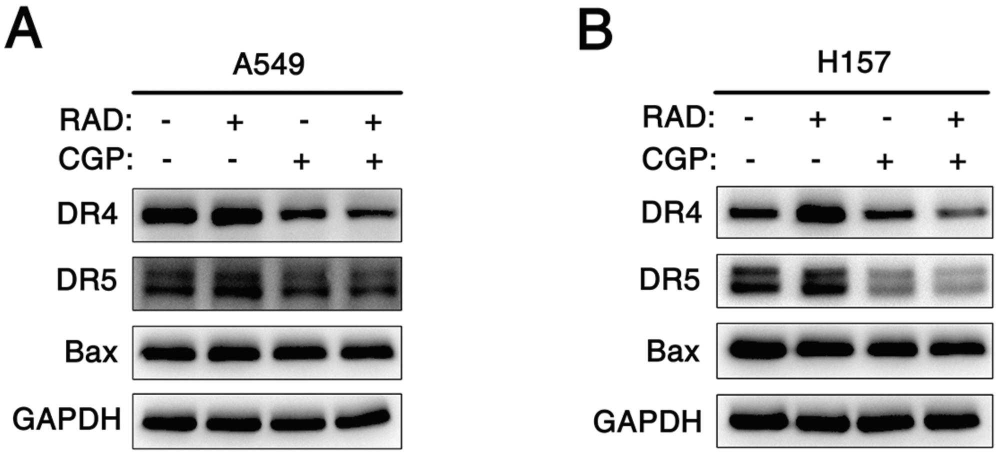

Supplementary Figure S1: Detection of DR4, DR5 and Bax levels in A549 and H157 cells. Western blotting analysis of DR4, DR5 and Bax in A549 **A,** and H157 **B.** Cells were treated with 25nM RAD001 (RAD) alone, 25μM CGP57380 (CGP) alone and their combination for 24 h. GAPDH severed as a loading control.

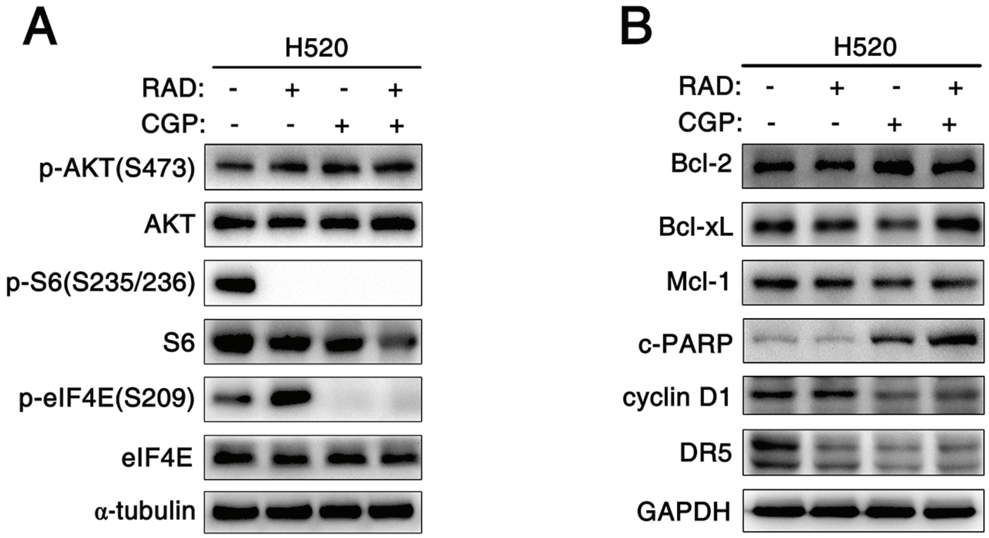

Supplementary Figure S2: Mnk1 inhibitor CGP57380 abrogates the eIF4E phosphorylation induced by mTOR inhibitor RAD001 and the combination of CGP57380 and RAD001 elicites apoptosis in H520 cells. A-B. H520 cells were treated with 25nM RAD001 (RAD) alone, 25μM CGP57380 (CGP) alone and their combination for 24 h. Then cell lysates were collected and subjected to western blotting for the indicated proteins. α-tubulin or GAPDH was used as a loading control.

Supplementary Table S1: Clinicopathological characteristics of studied 353 cases of non-small cell lung cancer (NSCLC) patients

Patients characteristics	No. of patients (%)
**Age(years)**	
≤50	98(27.8)
>50	255(72.2)
**Gender**	
Male	267(75.6)
Female	86(24.4)
**Clinical stages**	
Stage I	78(22.1)
Stage II	72(20.4)
Stage III	169(47.9)
Stage IV	34(9.6)
**Lymph node status**	
N0	142(40.2)
N1/N2/N3	211(59.8)
**Histological type**	
SCC	159(45.0)
ADC	194(55.0)
**Pathological grades**	
Well	6(1.7)
Moderate	144(40.8)
Poor	203 (57.5)

Supplementary Table S2: Analysis of the association between expression of p-Mnk1 and p-eIF4E proteins and clinicopathological features of NSCLC (n=353)

Clinicopathological features (n)	p-Mnk1			p-eIF4E			p-Mnk1 / p-eIF4E#		
	P (%)	N (%)	P-value	P (%)	N (%)	P-value	P^+ (%)	N^- (%)	P-value
**Age(years)**									
≤50 (n=98)	71(72.4)	27(27.6)		48(49.0)	50(51.0)		46(46.9)	52(53.1)	
>50 (n=255)	161(63.1)	94(36.9)	0.099	138(54.1)	117(45.9)	0.387	114(44.7)	141(55.3)	0.706
**Gender**									
Male(n=267)	171(64.0)	96(36.0)		138(51.7)	129(48.3)		119(44.6)	148(55.4)	
Female(n=86)	61(70.9)	25(29.1)	0.242	48(55.8)	38(44.2)	0.505	41(47.7)	45(52.3)	0.615
**Clinical stages**									
$Stage_{I-II}$ (n=150)	92(61.3)	58(38.7)		79(52.7)	71(47.3)		63(42.0)	87(58.0)	
$Stage_{III-IV}$ (n=203)	140(69.0)	63(31.0)	0.135	107(52.7)	96(47.3)	0.994	97(47.8)	106(52.2)	0.281
**LN status**									
LNM (n=211)	141(66.8)	70(33.2)		115(54.5)	96(45.5)		102(48.3)	109(51.7)	
No LNM (n=142)	91(64.1)	51(35.9)	0.595	71(50.0)	71(50.0)	0.406	58(40.8)	84(59.2)	0.165
**Histological type**									
SCC(n=159)	95(59.7)	64(40.3)		67(42.1)	92(57.9)		57(35.8)	102(64.2)	
ADC(n=194)	137(70.6)	57(29.4)	0.032*	119(61.3)	75(38.7)	0.000*	103(53.1)	91(46.9)	0.001*
**Pathological grade**									
Well (n=6)	6(100.0)	0(0.0)		5(83.3)	1(16.7)		5(83.3)	1(16.7)	
Moderate(n=144)	93(64.6)	51(35.4)		72(50.0)	72(50.0)		63(43.8)	81(56.3)	
Poor (n=203)	133(65.5)	70(34.5)	0.200	109(53.7)	94(46.3)	0.252	92(45.3)	111(54.7)	0.162
**Survival status**									
Alive(n=186)	107(57.5)	79(42.5)		84(45.2)	102(54.8)		68(36.6)	118(63.4)	
Dead(n=167)	125(74.9)	42(25.1)	0.001*	102(61.1)	65(38.9)	0.003*	92(55.1)	75(44.9)	0.000*

*Chi-square test, statistically significant difference ($P < 0.05$). Abbreviations: LNM, lymph node metastasis; SCC, squamous cell carcinoma; P, Positive; N, Negative; #P^+, common positive

Supplementary Table S3: The pairwise association between expression of p-Mnk1 and p-eIF4E proteins in 353 cases of NSCLC by Spearman's rank correlation test

	p-Mnk1		
	**Positive (%)**	**Negative (%)**	***P*-value**
**p-eIF4E**			
Positive (%)	160(86.0)	26(14.0)	0.000
Negative (%)	72(43.1)	95(56.9)	(r = 0.451)

*Spearman's rank correlation test, statistically significant difference ($P < 0.05$)
